# Pregnancy Outcomes of Anti-Hypertensives for Women with Chronic Hypertension: A Population-Based Study

**DOI:** 10.1371/journal.pone.0053844

**Published:** 2013-02-06

**Authors:** Chen-Yi Su, Herng-Ching Lin, Hsin-Chung Cheng, Amy Ming-Fang Yen, Yi-Hua Chen, Senyeong Kao

**Affiliations:** 1 Graduate Institute of Life Sciences, National Defense Medical Center, Taipei, Taiwan; 2 School of Health Care Administration, Taipei Medical University, Taipei, Taiwan; 3 School of Dentistry, College of Oral Medicine, Taipei Medical University, Taipei, Taiwan; 4 Department of Dentistry, Taipei Medical University Hospital, Taipei, Taiwan; 5 School of Oral Hygiene, College of Oral Medicine, Taipei Medical University, Taipei, Taiwan; 6 School of Public Health, Taipei Medical University, Taipei, Taiwan; 7 School of Public Health, National Defense Medical Center, Taipei, Taiwan; National Institutes of Health - National Institute of Child Health and Human Development, United States of America

## Abstract

**Background:**

The impact of anti-hypertensive treatment on fetus was unclear, and hence, remains controversial. We set out in this study to estimate the prevalence of adverse pregnancy outcomes, including low birth weight, preterm delivery and small for gestational age amongst women with chronic hypertension, and to determine whether the use of anti-hypertensive drugs increases the risk of such adverse pregnancy outcomes.

**Methodology/Principal Findings:**

A total of 2,727 hypertension mothers and 8,181 matched controls were identified from the population-based cohort. These hypertension women were divided into seven sub-groups according to different types of prescribed anti-hypertensive drugs. Multivariable logistic regressions were conducted to estimate the risk of low birth weight, preterm birth and small for gestational age. Increased risk of low birth weight (OR = 2.29, 95% CI = 1.95–2.68), preterm birth (OR = 2.18, 95% CI = 1.89–2.52) and small for gestational age (OR = 1.62, 95% CI = 1.45–1.81) were all discernible within the hypertension group after adjusting for potential confounding factors. The increased ORs were found to differ with different types of anti-hypertensive drugs. Women who received vasodilators were associated with the highest risk of low birth weight (OR = 2.96, 95% CI = 2.06–4.26), preterm birth (OR = 2.92 95% CI = 2.06–4.15) and small for gestational age (OR = 2.12, 95% CI = 1.60–2.82).

**Conclusions/Significance:**

This finding is important for practitioners, because it indicates the need for caution while considering the administration of anti-hypertensive drugs to pregnant women. These observations require confirmation in further studies that can better adjust for the severity of the underlying HTN.

## Introduction

Chronic hypertension, which is found to occur in approximately one to three per cent of all pregnancies, is one of the leading causes of maternal mortality [1]–[3]. Anti-hypertensive treatment has been proven to prevent, or delay, the occurrence of serious complications during pregnancy. Furthermore, it was reported in a recent Cochrane review that anti-hypertensive treatment reduces the risk of developing severe hypertension and is also beneficial to pregnant women. However, an increasing trend for small-for-gestational-age (SGA) babies was also discernible amongst mothers who had received anti-hypertensive treatment. The review concluded by pointing out that the impact of anti-hypertensive treatment on babies was still unclear [4], and hence, remains controversial.

Some of the prior studies have demonstrated that the maternal use of anti-hypertensive treatment could improve pregnancy outcomes, with one particular study indicating that early anti-hypertensive treatment was associated with a reduced probability of premature delivery [5], and another demonstrating a trend towards higher birth weights of babies in those cases where maternal anti-hypertensive treatment was used [6].

On the other hand, however, there are also several studies in which it is argued that such use of anti-hypertensive treatment can lead to adverse pregnancy outcomes; for example, one meta-analysis revealed that anti-hypertensive treatment may result in fetal growth restriction, and ultimately, in low-birth-weight (LBW) babies [7].

A higher risk of premature delivery, LBW and SGA infants amongst women receiving anti-hypertensive treatment has also been observed on data from the Swedish Medical Birth Registry [8]. Nevertheless, other studies have still been unable to determine any significant differences between the pregnancy outcomes of treated and untreated pregnant women [9]–[12].

Hampered by inadequate sample size, none of the prior studies have been able to provide a reliable estimation of the risk in having preterm, LBW or SGA births between different types of anti-hypertensive treatment.

In this study, we carry out a population-based cohort to address these undetermined issues, retrospectively. Our primary aim is to explore the association between pregnancy outcomes and maternal use of anti-hypertensive treatment, including different types of drugs, during the course of their pregnancy.

## Methods

### Data

The data examined in the present study are obtained from two national databases, the National Health Insurance Research Dataset (NHIRD) and the birth certificate registry. The NHIRD covers over 98 per cent of Taiwan’s 23 million citizens and contains comprehensive registration files and original claims data.

The birth certificate registry, obtained from the Department of Health in Taiwan, contains birthdates of both infants and parents, as well as details on gestational weeks at birth, birth weight, gender, parity, place of birth, and parental educational level. Since the registration of all births is mandatory in Taiwan, the birth certificate data is considered to be extremely accurate and comprehensive.

### Ethics Statement

The information contained within the NHIRD and the birth certificate registry is linked by unique personal identification numbers for the mothers and infants. All personal identifiers are encrypted by the Bureau of National Health Insurance before being released to researchers. Whilst the NHIRD consists of de-identified secondary data released to the public for research purposes, this study is exempt from a full review by the Internal Review Board.

### Study Sample

Our study covers the period from 1 January 2005 to 31 December 2005, from which we examined a population-based cohort comprising of all pregnant women in Taiwan. If any woman had more than one pregnancy during the study period, only the first pregnancy was considered for analysis and only singletons were included. The final sample comprised of a total of 218,781 mothers and 218,781 infants.

With considering that the presence of pre-eclampsia could be a major intermediary in the pathway between hypertension and preterm birth, we excluded 2,721 women diagnosed with pre-eclampsia (ICD-9-CM codes 642.5 and 642.6) and women diagnosed with gestational HTN (ICD-9-CM codes 642). Pregnant women who had been diagnosed as chronic hypertension (HTN) prior to their pregnancy were identified as the HTN group (n = 2,727), with the definition of chronic HTN being based upon the presence of ICD-9-CM codes 401–405 at either outpatient diagnosis or inpatient discharge diagnosis.

Although new data in the literature suggested that the teratogenicity of angiotensin-converting-enzyme (ACE) inhibitors is not larger than that of other antihypertensive drugs [8], the use of ACE inhibitors or Angiotensin II receptor blockers (ARBs) during pregnancy was still a controversial issue. Furthermore, due to the possible increased risk of major malformations raised by published studies [13], [14], ACE inhibitors and ARBs are contraindicated during pregnancy in Taiwan. These prescriptions would have been discontinued once a woman became aware of pregnancy. Therefore, we have excluded 120 women using ACE inhibitors or ARBs for further analysis. Women who used more than one type of anti-hypertensive drugs were also excluded (n = 722).

The HTN group was further divided into seven sub-groups in order to facilitate our examination of the impact on different types of anti-hypertensive drugs. One of these sub-groups was an untreated group (comprising of those within the HTN group who had not used any anti-hypertensive drugs); the remainder were separated into six sub-groups according to the different types of prescribed anti-hypertensive drugs used.

Data on anti-hypertensive drugs use come from NHIRD, which consisted of all claims data during pregnancy. Among HTN women, exposure to anti-hypertensive drugs was calculated from the date of having pregnancy to the date of giving birth. The date of having pregnancy means the conception date. The definition of drug-treated hypertension was made according to the days of prescribed anti-hypertensive treatment. Thus, in order to be considered as having undergone anti-hypertensive treatment, HTN women must have received a prescription of an anti-hypertensive drug for a period of at least 30 days during any time of their pregnancy. Anti-hypertensive drugs in this study are further categorized into six types, including central *α* agonists, beta-blockers (BBs), combined *α* and BBs, calcium channels blockers, diuretics and vasodilators. The drug group made up of combined <alpha> agonists and <beta> blockers means that the drug blocked the binding to both <alpha> and <beta>receptors, for example, carvedilol and labetalol. Women who used more than one type of anti-hypertensive drugs were excluded from the analysis.

**Figure 1:**
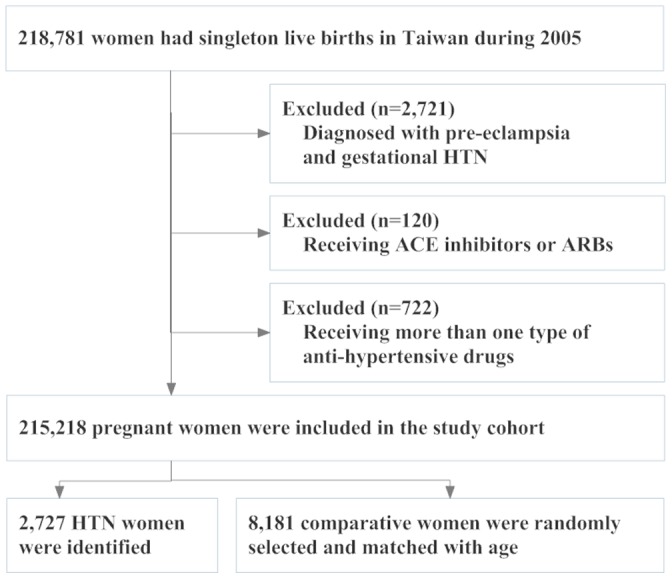
Diagram of the Population-Based Study.

For each HTN woman, three women with no diagnosis of chronic HTN were randomly selected from the same cohort, matched with both age and year of delivery. As a result of this matching process, the study sample ultimately comprised of a total of 2,727 pregnant women with chronic HTN, and 8,181 pregnant women with no chronic HTN (as the comparison group) for analysis. The study flow diagram was shown in Figure 1.

### Variables of Interest

The focus in this study is on the three pregnancy outcomes of low birth weight (LBW), preterm birth and small for gestational age (SGA). The primary outcome variable, LBW, is defined in accordance with the World Health Organization criterion, as birth weight <2,500 gm. Our secondary outcome variable, preterm birth is defined as gestational duration <37 completed weeks, and the final outcome variable, SGA, is defined as <10^th^ percentile for gestational age and gender [15].

Covariates which might potentially influence the outcomes of interest were also considered. Infant demographic characteristics included parity, whilst maternal characteristics included age, the highest educational level, and comorbid conditions, comprising of diabetes (ICD-9-CM codes 250), anemia (ICD-9-CM codes 280–289), coronary heart disease (ICD-9-CM codes 410–414, 429) and hyperlipidemia (ICD-9-CM codes 272).

### Statistical Analysis

Pearson Chi-squared (*χ* ^2^) tests were used to compare the differences in demographic factors: (i) between the HTN and comparison groups, (ii) among the seven subgroups receiving various types of anti-hypertensive drugs and the untreated group, and (iii) among the five subgroups receiving various types of anti-hypertensive drugs. The effects of chronic HTN on pregnancy outcomes were examined with conditional logistic regression models to estimate the risk of LBW, preterm birth and SGA for women with chronic HTN. We then employed logistic regression analyses amongst the HTN group only to evaluate the risk of LBW, preterm birth and SGA associated with the use of various anti-hypertensive drugs, as compared to untreated women. The previously mentioned covariates, which might potentially confound the pregnancy outcomes, were also included in the multivariable analysis. Odds ratios (ORs) and 95 per cent confidence intervals (95% CIs) were given. The analyses were undertaken using SAS version 9.1 software (SAS Institute, Cary, NC), with a two-sided *p*-value of <0.05 being considered to be statistically significant.

## Results

The maternal and infant characteristics of the HTN group and the age-matched comparison group are compared in Table 1; HTN mothers were more likely to have a diagnosis of diabetes, anemia, coronary heart disease and hyperlipidemia (*p*<0.001). Differences between the mothers with normotension and HTN, in terms of infant parity, and maternal education level, were also found to be statistically significant (*p*<0.001).

**Table 1:** Mothers with Hypertension and Comparative Cohort, by Infant and Maternal Characteristics.

Variables	Mothers with Hypertension n = 2,727		Comparative Cohort n = 8,181		*P* value
	Total no.	%	Total no.	%	
Infant characteristics					
Gender					0.127
Male	1,393	51.1	4,317	52.8	
Parity					<0.001
1	1,303	47.8	4,132	50.5	
2	939	34.4	3,039	37.2	
3 or more	485	17.8	1,010	12.4	
Gestational age					<0.001
Weeks (mean±SD)	37.9±1.9		38.5±1.5		
Birthweight					<0.001
G (mean±SD)	3052±545		3131±423		
Maternal characteristics					
Age (year)					
<25	310	11.4	930	11.4	
25–29	842	30.9	2,526	30.9	
30–34	916	33.6	2,748	33.6	
>34	659	24.1	1,977	24.1	
Education level					<0.001
Elementary school or below	406	14.9	862	10.5	
Junior high school	1,140	41.8	3,031	37.1	
Senior high school	1,096	40.2	3,928	48.0	
College or above	85	3.1	360	4.4	
Diabetes					<0.001
Yes	226	8.3	186	2.3	
Anemia					<0.001
Yes	314	11.5	652	8.0	
Coronary heart disease					<0.001
Yes	121	4.4	49	0.6	
Hyperlipidemia					<0.001
Yes	179	6.6	95	1.2	

Table 2 compares maternal and infant characteristics between the six sub-groups of different anti-hypertensive drug types and comparative cohort. Statistically significant differences were found in maternal characteristics, such as age, education level, diabetes, anemia, coronary heart disease, hyperlipidemia and infant parity between the sub-groups (*p*<0.05), but not for infant gender. Similar results were shown in the additional chi-square test for comparison between the six sub-groups in the HTN.

**Table 2:** Mothers with Hypertension, by Infant and Maternal Characteristics and Type of Anti-Hypertensive Drug.

Variables	Diuretics n = 371		BBs n = 414		CCBs n = 303		Central α agonists n = 181		Combined α and BBs n = 123		Vasodilators n = 329		None n = 1,006		*P* value	*P* value-2
	No.	%	No.	%	No.	%	No.	%	No.	%	No.	%	No.	%		
Infant characteristics																
Gender															0.999	0.999
Male	191	51.5	211	50.9	155	51.2	90	49.7	61	49.6	167	50.8	518	51.5		
Parity															<0.001	<0.01
1	195	52.6	169	40.8	164	54.1	96	53.0	65	52.9	177	53.8	437	43.4		
2	109	29.3	154	37.2	102	33.7	51	28.2	40	32.5	103	31.3	380	37.8		
3 or more	67	18.1	91	22.0	37	12.2	34	18.8	18	14.6	49	14.9	189	18.8		
Gestational age															<0.001	<0.001
Weeks (mean±SD)	38.1±1.9		37.9±1.7		37.8±1.9		37.5±2.1		37.7±1.9		37.4±2.3		38.1±1.8			
Birthweight															<0.001	<0.001
G (mean±SD)	3107±507		3051±499		3047±545		2951±667		2962±565		2908±656		3109±499			
Maternal characteristics																
Age (years)															<0.001	<0.001
<25	64	17.2	51	12.3	42	13.9	19	10.5	9	7.3	27	8.2	98	9.7		
25–29	116	31.3	127	30.7	105	34.6	49	27.1	26	21.2	100	30.4	319	31.7		
30–34	124	33.4	123	29.7	93	30.7	65	35.9	49	39.8	113	34.3	349	34.7		
>34	67	18.1	113	27.3	63	20.8	48	26.5	39	31.7	89	27.1	240	23.9		
Education level															<0.01	<0.05
Elementary school or below	69	18.6	67	16.2	44	14.5	31	17.1	12	9.8	50	15.2	133	13.2		
Junior high school	153	41.2	198	47.8	117	38.6	82	45.3	47	38.2	128	38.9	415	41.3		
Senior high school	142	38.3	139	33.6	130	42.9	66	36.5	58	47.2	146	44.4	415	41.3		
College or above	7	1.9	10	2.4	12	4.0	2	1.1	6	4.9	5	1.5	43	4.3		
Diabetes															<0.001	<0.01
Yes	11	3.0	25	6.0	32	10.6	19	10.5	9	7.3	25	7.6	105	10.4		
Anemia															<0.001	<0.01
Yes	67	18.1	51	12.3	40	13.2	19	10.5	9	7.3	27	8.2	101	10.0		
Coronary heart disease															<0.01	<0.001
Yes	8	2.2	33	8.0	11	3.6	2	1.1	6	4.9	14	4.3	47	4.7		
Hyperlipidemia															<0.01	0.304
Yes	13	3.5	24	5.8	16	5.3	11	6.1	10	8.1	13	4.0	92	9.2		

BBs indicates beta-blockers; CCBs, calcium channels blockers.

*P* value and *P* value-2 are estimated from chi-square test; *P* value-2 is not including none-treated group.

The distribution and odds ratios of LBW, preterm birth and SGA, for women with and without chronic HTN, are presented in Table 3. Within the HTN group, incidences of LBW, preterm birth and SGA infants were significantly higher (*p*<0.001). Increased risk of LBW (OR = 2.37, 95% CI = 2.03–2.76), preterm birth (OR = 2.26, 95% CI = 1.97–2.61) and SGA (OR = 1.63, 95% CI = 1.47–1.82) were also discernible amongst HTN women.

**Table 3:** ORs of LBW, Preterm Birth and SGA amongst Mothers with Hypertension and Comparatives.

Variables	Mothers with Hypertension n = 2,727		Comparative Cohort n = 8,181		Mothers with Untreated Hypertension n = 1,006	
	Total no.	%	Total no.	%	Total no.	%
LBW						
Yes	308	11.3	418	5.1	76	7.6
Crude OR	2.37		1.00		1.52	
(95% CI)	(2.03–2.76)‡				(1.18–1.97)†	
Adjusted OR§	2.29		1.00		1.47	
(95% CI)	(1.95–2.68)‡				(1.13–1.91)†	
Preterm Birth						
Yes	369	13.5	529	6.5	84	8.4
Crude OR	2.26		1.00		1.32	
(95% CI)	(1.97–2.61)‡				(1.04–1.68)*	
Adjusted OR§	2.18		1.00		1.30	
(95% CI)	(1.89–2.52)‡				(1.01–1.66)*	
SGA						
Yes	620	22.7	1250	15.3	184	18.3
Crude OR	1.63		1.00		1.24	
(95% CI)	(1.47–1.82)‡				(1.05–1.47)*	
Adjusted OR§	1.62		1.00		1.27	
(95% CI)	(1.45–1.81)‡				(1.07–1.52)*	

OR indicates odds ratio; LBW, low birth weight; SGA, small for gestational age; CI, confidence interval.

* *P*<0.05;

† *P*<0.01;

‡ *P*<0.001;

§ Adjusted for infant parity, maternal age, education level, diabetes, anemia, coronary heart disease, and hyperlipidemia.

After adjusting for the mother’s parity, maternal age, education level, diabetes, anemia, coronary heart disease, and hyperlipidemia, the adjusted odds ratios of LBW, preterm birth and SGA were 2.29 (95% CI = 1.95–2.68), 2.18 (95% CI = 1.89–2.52) and 1.62 (95% CI = 1.45–1.81) higher for HTN women.

The crude and adjusted ORs of preterm birth, LBW, and SGA for non-treated HTN mothers (n = 1,006), compared to non-HTN controls (n = 8,181) were shown in Table 3 to reveal the “pure” HTN effect.

The distribution and odds ratios of LBW, preterm birth and SGA, relating to the use of various anti-hypertensive drug types are presented in Table 4. A higher frequency and an increased risk of LBW, preterm birth and SGA were discernible amongst women treated with anti-hypertensive drugs, as compared to women who were untreated, with these differences all being found to be statistically significant.

**Table 4:** ORs for Mothers with Hypertension Having LBW, Preterm Births or SGA, by Type of Anti-Hypertensives.

Variables	Diuretics n = 371		BBs n = 414		CCBs n = 303		Central α agonists n = 181		Combined α and BBs n = 123		Vasodilators n = 329		None n = 1,006		*P* value	*P* value-2
	No.	%	No.	%	No.	%	No.	%		%	No.	%	No.	%		
LBW															<0.001	<0.001
Yes	32	8.6	52	12.6	32	10.6	34	18.8	19	15.5	63	19.2	76	7.6		
Crude OR	1.16		1.76		1.45		2.83		2.24		2.90		1.00			
(95% CI)	(0.75–1.78)		(1.21–2.55)†		(0.94–2.23)		(1.82–4.40)‡		(1.30–3.84)†		(2.02–4.16)‡					
Adjusted OR§	1.16		1.70		1.50		2.88		2.22		2.96		1.00			
(95% CI)	(0.75–1.80)		(1.17–2.48)†		(0.97–2.33)		(1.85–4.49)‡		(1.29–3.84)†		(2.06–4.26)‡					
Preterm Birth															<0.001	0.076
Yes	48	12.9	65	15.7	47	15.5	37	20.4	20	16.3	68	20.7	84	8.4		
Crude OR	1.63		2.04		2.02		2.82		2.13		2.86		1.00			
(95% CI)	(1.12–2.38)*		(1.45–2.89)‡		(1.37–2.96)‡		(1.84–4.31)‡		(1.26–3.62)†		(2.02–4.05)‡					
Adjusted OR§	1.73		2.01		2.11		2.87		2.13		2.92		1.00			
(95% CI)	(1.18–2.53)†		(1.42–2.86)‡		(1.43–3.10)†		(1.87–4.40)‡		(1.25–3.63)†		(2.06–4.15)‡					
SGA															<0.001	<0.001
Yes	62	16.7	103	24.9	74	24.4	53	29.3	39	31.7	105	31.9	184	18.3		
Crude OR	0.90		1.48		1.44		1.85		2.07		2.09		1.00			
(95% CI)	(0.65–1.23)		(1.13–1.95)*		(1.06–1.96)*		(1.29–2.65)‡		(1.37–3.13)‡		(1.58–2.78)‡					
Adjusted OR§	0.89		1.44		1.45		1.88		2.11		2.12		1.00			
(95% CI)	(0.65–1.22)		(1.09–1.90)†		(1.06–1.97)*		(1.31–2.70)‡		(1.39–3.19)‡		(1.60–2.82)‡					

OR indicates odds ratio; LBW, low birth weight; SGA, small for gestational age; CI, confidence interval; BBs, beta-blockers; CCBs, calcium channels blockers.

*P* value and *P* value-2 are estimated from chi-square test; *P* value-2 is not including none-treated group.

* *P*<0.05;

† *P*<0.01;

‡ *P*<0.001;

§ Adjusted for infant parity, maternal age, education level, diabetes, anemia, coronary heart disease, and hyperlipidemia.

The increased ORs were also found to differ with the treatment groups and untreated group. As regards the risk of LBW, vasodilators had the greatest crude and adjusted ORs (2.90, 95% CI = 2.02–4.16; 2.96, 95% CI = 2.06–4.26), followed by central α agonists (2.83, 95% CI = 1.82–4.40; 2.88, 95% CI = 1.85–4.49).

As for preterm birth, vasodilators had the greatest crude and adjusted ORs (2.86, 95% CI = 2.02–4.05; 2.92, 95% CI = 2.06–4.15), followed by central α agonists (2.82, 95% CI = 1.84–4.31; 2.87, 95% CI = 1.87–4.40), whilst diuretics had the lowest crude and adjusted ORs (1.63, 95% CI = 1.12–2.38; 1.73, 95% CI = 1.18–2.53). The greatest crude and adjusted ORs of SGA were found in vasodilators (2.09, 95% CI = 1.58–2.78; 2.12, 95% CI = 1.60–2.82), followed by combined *α* and BBs (2.07, 95% CI = 1.37–3.13; 2.11, 95% CI = 1.39–3.19).

## Discussion

The population-based cohort adopted for this study revealed that infants born to women with chronic HTN were at an increased risk of LBW, preterm birth and SGA. Our results further demonstrate that all types of anti-hypertensive drugs are associated with significantly higher risks of such adverse pregnancy outcomes amongst women with chronic HTN, at varying levels. The greatest risk for such adverse pregnancy outcomes was found in the use of vasodilators, as compared to other anti-hypertensive drugs. The relatively safer types of anti-hypertensive drugs were found to be BBs and CCBs. We have further investigated the trimester of pregnancy for receiving anti-hypertensive treatments among HTN mothers and found most HTN women (80.8%) receiving anti-hypertensive treatments from the first trimester of pregnancy.

The prevalence of chronic HTN observed in this study, at 1.2 per cent of all pregnancies fall within the range of 1 to 3 per cent reported in earlier studies in other countries [1], [3], [8], [16]. The findings of this study indicate that, for women with chronic HTN, the increased risks of 2.29 for LBW, 2.18 for preterm delivery, and 1.62 for SGA in Taiwan are within the range of 1.5–8.5 for LBW, [17], [18] 1.4–2.4 for preterm delivery, [17], [19] and 1.5–2.0 for SGA, [17], [20], [21] reported in the prior studies which were conducted in western countries. [17]–[21] There is a comprehensive study from the Swedish Medical Birth Register [8]. The ORs for preterm birth, LBW, and SGA are 4.72 for LBW, 3.33 for preterm delivery, and 4.23 for SGA, higher than those described in the present manuscript.

The prevalence of pre-existing type I/II diabetes mellitus in our study was 2.3% in comparative cohort, which was similar with the 3.2% previously reported in Taiwan [22], [23], but higher than the rate of 0.4% in the north of England [24], 0.4% to 0.7% in Australia [25] and 1.4% reported from Swedish data [8]. In Taiwan, the prevalence of diabetes has almost doubled between 1985 and 1996 [26]. The increasing number of diabetes population could result in the high proportion of diabetes for pregnant women in Taiwan. Due to the different effects on pregnancy, we have further investigated the types of diabetes and found mothers with type 1 diabetes (ICD-9-CM codes 250.×1 and 250.×3) only accounted for 0.05% of all pregnancies (116 of 218,781).

In our study, the diagnosis of diabetes was based on ICD-9-CM codes 250, including type 1 and type 2, which had been stated in Page 10, Line 168. The Swedish data in ref 8 was also using the diagnosis of diabetes to determine whether the presence of diabetes or not. Ref 24 and ref 25 used the different method to collect the disease condition. Data from ref 24 presented the survey records of 1,677 type 1 and type 2 diabetes women among 401,149 singleton pregnancies between 1996 and 2008. Data from ref 25 was collected data using standardized measures and showed the 0.4%, 0.6% and 0.7% of pre-existing diabetes, including type 1 and type 2, among women aged 25–29, 35–39 and >39 years between 1998 and 2008. The different proportion of diabetes could be due to the different method used to collect the disease condition.

We observed a higher proportion of infants classified as SGA (15.3%) in comparative cohort than the nominal 10%. The rate of SGA in our study is similar to the 15.2% to 16.7% previously reported in Taiwan using the same definition way [22], . Obviously, the 10th percentile should refer to the birth weight distribution in each weak, term or preterm. The reference used [15] was published in 1991 and is not adequate for the present population. This does not affect comparisons between the seven HTN groups. To the best of our knowledge, this is the first study to focus on different types of anti-hypertensive drugs as a means of investigating subsequent pregnancy outcomes within the same chronic HTN cohort; indeed, the majority of the prior studies have tended to be conducted on small samples, emphasizing the specific LBW effect of BBs [28]–[30].

Our findings are in line with the results of several prior published works [28], [29], [31]. In a small clinical trial by Butters et al., mothers who were exposed to BBs had significantly more LBW infants than those treated with a placebo [28]. Lydakis et al. reported that BBs were not only associated with LBW, since they also detected higher prevalence of both preterm delivery and SGA infants in the BBs group [29]. Ray et al. similarly suggested that HTN mothers who had received BBs were at an increased risk of both preterm births (OR = 4.0) and SGA babies (OR = 2.3) [31].

The information currently available on pregnancy outcomes relating to the effects of other types of anti-hypertensive drugs is rather limited. Our observation of increased LBW, preterm and SGA risks for HTN women exposed to CCBs or diuretics is in line with the three studies referred to above [32]–[34].

A trial conducted by Fenakel et al. revealed that infants born to women treated with CCBs tended to have more advanced gestational ages and increased weight [33]. This result provides support for our finding that, amongst the seven types of anti-hypertensive drugs, CCBs present the smallest risk of LBW.

One possible explanatory mechanism for the increased risk of delivering LBW infants amongst women using anti-hypertensive drugs is the treatment associated with intra-uterine growth retardation. In the meta-analysis referred to earlier, it was noted that for a 10 mmHg reduction in maternal blood pressure induced by anti-hypertensive treatment, there was a potential corresponding reduction of 145 gm in fetal growth [7].

In contrast to the results of the present study, few randomized trials failed to show any difference in average birth weight, prevalence of preterm birth or SGA between women treated with anti-hypertensive drugs and untreated HTN women, as did one systematic review [10], [11], [30], [35]–[37]; however, as compared to the sample size in the present study, relatively small samples were involved in each of these studies. Three randomized trials carried out on samples ranging from 25 to 242 HTN women reported that the infant birth weights for those women prescribed with central *α* agonists and those in the placebo group were very similar [35], [36].

The particular strengths of the present study are its large sample size, the valuable socioeconomic information available from the data source, and the unique ability to assess anti-hypertensive drug use with computerized pharmacy records. The computerized pharmacy records from NHIRD contain all medical claims data of pregnant women. In Taiwan, approximately 99% of all Taiwanese citizens joined in the National Health Insurance program. The NHIRD therefore has the comprehensive medical records. Our study is clearly well positioned to provide more reliable results given the large sample size, and since the study involves a population-based dataset which enables us to study all of the pregnancies in Taiwan, we can clearly minimize selection bias, as well as other preferred prescription biases.

The database adopted for this study involved several important socio-economic factors, such as maternal education level, and marital status, all of which may potentially affect the association observed between anti-hypertensive drugs and adverse pregnancy outcomes; adjustments were made for these potential confounders within the multivariable analysis. Furthermore, as opposed to reliance on self-reported data, which can give rise to doubts as to the accuracy of the type of anti-hypertensive drugs reportedly used, the comprehensive pharmacy database allows precise assessment of the anti-hypertensive drugs prescribed.

There are several limitations of the present study which should be taken into consideration. Given that we were unable to assess whether or not patients actually took the drugs that they were prescribed, adherence to medication remains unknown; nevertheless, given that patients are required to pay a proportion of their medication charges, there would be an increased likelihood of them actually using the prescribed anti-hypertensive drugs. It is very likely that HTN mothers choose not to take the prescribed anti-hypertensives due to the worries of possible hazards for their babies. In this case, our analysis may give conservative results. If mothers take all prescribed anti-hypertensive drugs, the risk could be higher than what we estimated in the study.

A second limitation is that details on certain covariates, such as maternal smoking, diet, obesity and the use of alternative non-prescription drugs, are not available from the administrative database. Although the current study used maternal comorbidity as possible confounders, further study incorporating maternal smoking, BMI and other possible confounders is needed to validate findings of comparison between HTN and comparative cohort from present study. Nevertheless, we believe that maternal smoking, diet, and obesity will not distribute unbalanced across the different treatment sub-groups. The impact of ignorance of these factors in the comparison between specific anti-hypertensives among HTN pregnant mother would be small. The final limitation noted here is that we had no access to patient records which would allow us to adjust for maternal blood pressure, a factor which represents the clinical severity of HTN. However, we could assume the women’s blood pressure was well-controlled under anti-hypertensive treatment. The effect of original severity of HTN would be minimized with anti-hypertensive treatment. The severity of the underlying HTN may be the cause of the found deviations and the differences between the drug treated groups may also be caused by different severity of the HTN. So, for instance it seems likely that women who only got diuretics have a milder form than women who got other anti-hypertensive drugs. The confounding by indication could not be fully excluded from our study and one should take these limitations with our results into consideration.

In conclusion, our study demonstrates that those women with chronic HTN had an increased risk of preterm delivery, LBW and SGA infants. Furthermore, our data suggests that mothers treated with anti-hypertensive drugs were at a greater risk of these adverse pregnancy outcomes than untreated mothers, and it seems likely that women who only got diuretics have a milder form than women who got other anti-hypertensive drugs. Our findings demonstrate that the association between the maternal use of anti-hypertensive drugs and infants with LBW and SGA is not specific to BBs, but is also discernible amongst other types of anti-hypertensive drugs. This finding is important for practitioners, because it indicates the need for caution while considering the administration of anti-hypertensive drugs to pregnant women. These observations require confirmation in further studies that can better adjust for the severity of the underlying HTN.
